# Perkutane Leberbiopsie vor Organentnahme – Einfluss auf Organallokation und Kosten in der Lebertransplantation

**DOI:** 10.1007/s00104-020-01192-w

**Published:** 2020-05-19

**Authors:** Christian Beltzer, Markus Quante, Myriam Rheinberger, Hideo Andreas Baba, Fuat Saner, Falko Fend, Thomas Biet, Alfred Königsrainer, Silvio Nadalin

**Affiliations:** 1 Klinik für Allgemeine, Viszeral- und Transplantationschirurgie, Universitätsklinikum Tübingen, Tübingen, Deutschland; 2 Institut für Pathologie, Universitätsklinikum Essen, Essen, Deutschland; 3 Klinik für Allgemein‑, Viszeral- und Transplantationschirurgie, Universitätsklinikum Essen, Essen, Deutschland; 4 Institut für Pathologie und Neuropathologie, Universitätsklinikum Tübingen, Tübingen, Deutschland; 5 Deutsche Stiftung Organtransplantation, Frankfurt am Main, Deutschland

**Keywords:** Kalte Ischämiezeit, Lebersteatose, Erweiterte Spenderkriterien, Spenderevaluation, Cold ischemia time, Liver steatosis, Extended donor criteria, Donor evaluation

## Abstract

**Hintergrund:**

Der Stellenwert und die Sicherheit einer perkutanen Leberbiopsie (PLB) bei hirntoten Spendern vor Organentnahme sowie der Einfluss der PLB auf die Organallokation und die Kosten im Rahmen der Lebertransplantation (LT) in der Eurotransplant-Region (ET), werden weiterhin diskutiert.

**Material und Methoden:**

Eine perkutane Leberbiopsie vor Organentnahme erfolgte bei 36 hirntoten Spendern. Die Komplikationsrate, Spendercharakteristika, Ultraschallbefunde, die makroskopische Einschätzung und die histologischen Ergebnisse der PLB wurden analysiert. Zusätzlich wurde eine landesweite Umfrage unter 11 Lebertransplantationsexperten durchgeführt. Der Bedarf einer PLB und ihre Auswirkungen auf den Prozess der Organallokation wurden evaluiert. Mögliche Kosteneinsparungen wurden für verschiedene Szenarien auf der Grundlage von Kostendaten der Deutschen Stiftung Organtransplantation berechnet.

**Ergebnisse:**

Es wurden keine Komplikationen durch die PLB beobachtet. Die Umfrage ergab, dass das Ergebnis der PLB einen erheblichen Einfluss auf die Allokation von Spenderorganen hat, insbesondere bei solchen mit „extended donor criteria (EDC)“. Die Kostenberechnung ergab ein enormes Kosteneinsparungspotenzial durch eine optimierte Allokation und die Vermeidung unnötiger Organentnahmen.

**Schlussfolgerung:**

Die PLB ist ein sicheres Verfahren und besitzt ein enormes Potenzial für die Optimierung der Organallokation vor Organentnahme durch eine Reduzierung der kalten Ischämiezeit, Vermeidung unnötiger Verwerfungen von Spenderorganen sowie Kosteneinsparungen. Die klinische Relevanz und der Einfluss der PLB auf die Organallokation konnte durch unsere Daten herausgestellt werden.

## Abstract (en)

**Background:**

The value and safety of percutaneous liver biopsy (PLB) in brain-dead donors before organ removal and its impact on organ allocation and costs of liver transplantation (LT) in the Eurotransplant (ET) region is still a matter of ongoing debate.

**Material and methods:**

A PLB was performed in 36 brain-dead organ donors. The complication rate, ultrasonography findings, macroscopic evaluation and histological results of PLB and donor characteristics were analyzed. Additionally, a nationwide survey was conducted among 11 liver transplantation experts. The need for PLB and its impact on the liver allocation process were evaluated. Possible cost savings were calculated for different scenarios based on cost data provided by the German Organ Transplantation Foundation.

**Results:**

No complications of PLB were observed. The survey revealed that the PLB has a substantial impact on the allocation of donor organs, especially in organs fulfilling extended donor criteria (EDC). The cost calculation revealed an enormous potential for cost savings due to an optimized organ allocation process and avoidance of futile organ procurement.

**Conclusion:**

The PLB is a safe procedure and has tremendous potential for the optimization of the organ allocation process before organ procurement by reducing the cold ischemia time, avoiding unnecessarily discarding donor organs and saving costs. These data emphasize the clinical relevance and impact of PLB on the organ allocation process.

## Hintergrund

Die wachsende Diskrepanz zwischen dem hohen Bedarf und dem anhaltenden Mangel an verfügbaren Spenderorganen für Patienten mit „end-stage liver disease“ auf der Warteliste hat zu einer Erweiterung des Spenderpools geführt, indem vermehrt auf Spender mit erweiterten Spenderkriterien („extended criteria donors“, ECD) zurückgegriffen wird [1, 2].

Nach der Definition von Eurotransplant werden hirntote Spender („brain-dead donors“, BDD) mit ECD durch eines oder mehrere der folgenden 7 Kriterien charakterisiert („extended donor criteria“, EDC): 1) Spenderalter >65 Jahre, 2) Intensivstationsaufenthalt mit mechanischer Beatmung >7 Tage, 3) Body-Mass-Index (BMI) >30 kg/m^2^, 4) Lebersteatose >40 %, 5) Serumnatrium >165 mmol/l, 6) erhöhte Transaminasen (Alanin-Aminotransferase [ALT] > 1750 nmol/(s l) [> 105 U/l], Aspartat-Aminotransferase [AST] > 1500 nmol/(s l) [> 90 U/l]), 7) Serumbilirubin > 51.3 μmol/l [> 3 mg/dl] [1]. Innerhalb der Eurotransplant-Region beträgt der Anteil an Lebertransplantaten aus ECD >50 % [3], und es wird vermutet, dass er in Zukunft weiter ansteigen wird.

Funktion und Langzeitüberleben nach Lebertransplantation hängen stark von der Qualität des Spenderorgans ab, weshalb eine sorgfältige Bewertung und Auswahl desselben unerlässlich ist [4, 5]. Zur Einschätzung des Transplantatversagens in Abhängigkeit von den Spendereigenschaften haben Feng und Kollegen 2006 den „donor risk index“ (DRI) eingeführt [6], der 2012 auf die Eurotransplant-Region (ET-DRI) adaptiert wurde [7]. Innerhalb der Eurotransplantat-Region ist der durchschnittliche „MELD-Score“ („model of end stage liver disease“) der Empfänger zum Zeitpunkt der Transplantation nach Einführung des MELD-basierten Zuweisungssystems von 25 auf 34 gestiegen. Gleichzeitig hat die Qualität der Spenderorgane abgenommen, mit >60 % suboptimalen Organen mit einem DRI >1,5 Punkte. Dies hat zu schlechteren Ergebnissen und zu einer verminderten Überlebensrate nach Lebertransplantation (LT) geführt [8].

Parallel zur weltweiten Zunahme der Adipositas ist die Fettleberkrankheit („fatty liver diseaese“, FLD) zu einem bedeutenden Problem bei Lebertransplantationen geworden. Ihre Prävalenz wird mit Raten von 30 % bei Post-mortem-Spenden und 16–20 % bei Lebendleberspenden angegeben [9, 10]. Die Lebersteatose wird in drei Grade eingeteilt: mild (<30 % Steatose), moderat (≥30–60 % Steatose) und schwer (≥60 % Steatose; [11, 12]), wobei eine Steatose >40 % ein Merkmal der ECD ist [2]. Die nichtalkoholische Fettleberkrankheit („non-alcoholic fatty liver disease“, NAFLD) ist wiederum die Vorstufe der nichtalkoholischen Steatohepatitis (NASH), die bei Personen mit einem BMI ≥30–39 kg/m^2^ in bis zu 20 % und bei Personen mit einem BMI <30 kg/m^2^ in etwa 3 % der Fälle nachgewiesen werden kann [12]. Transplantationen von Spenderorganen mit milder Steatose weisen ähnliche 5‑Jahres-Überlebensraten wie nichtsteatotische Lebertransplantate auf [13]. Lebertransplantate mit moderatem Steatosegrad (≥30–60 %) sollten nur bei ausgewählten Empfängern [13–16] und Spenderorgane mit schwerer Steatose (≥60 % Steatose) sollten nicht transplantiert werden [1, 17]. Die makrovesikuläre Steatose ist ein unabhängiger Risikofaktor für das Transplantatüberleben, zusätzlich zu den im DRI beschriebenen Merkmalen [18]. Bemerkenswert in diesem Zusammenhang ist, dass ein deutsches Transplantationszentrum eine Zunahme stornierter Lebertransplantationen von 14,7 % im Jahr 2010 auf 63,6 % im Jahr 2016 aufgrund höhergradiger Lebersteatose berichtete [19].

Eine Laboruntersuchung vor Organentnahme ist zuverlässig zur Erkennung von Viruserkrankungen, aber weniger zuverlässig bei der Diagnose einer FLD. Die Serum-Gamma-Glutamyltransferase (GGT) ist ein empfindlicher Marker für die FLD, und erhöhte GGT-Spiegel sind mit einer fortgeschrittenen Fibrose bei der FLD assoziiert [20]. Die GGT ist jedoch als ein unspezifischer Marker anzusehen [21]. Der „FLD-Score“ (Alter, Hyperglykämie, BMI, Thrombozytenzahl, Albumin, AST/ALT-Verhältnis) erwies sich als zuverlässiges Instrument zur Vorhersage einer Fibrose bei FLD-Patienten [22]. Allerdings ist eine Steatose nicht unbedingt mit erhöhten Lebertransaminasen assoziiert [23], die in Bezug auf eine FLD eine niedrige Sensitivität aufweisen [24]. Zudem korrelieren biochemische Marker nicht mit dem Grad der Steatose [25].

In der klinischen Praxis wird die Ultraschalluntersuchung zur Detektion einer Lebersteatose (mild, moderat und schwer) eingesetzt. Die Ergebnisse und Reproduzierbarkeit der Sonographie unterliegen aber durchaus untersucherabhängigen Unterschieden [26]. Die histologische Untersuchung stellt daher immer noch den Goldstandard bei der Diagnose von Lebererkrankungen wie FLD, Fibrose und Entzündungen, aber auch bei der Graduierung von Lebersteatosen dar [1, 27–29]. Jedoch gehört die Leberbiopsie mit histologischer Befundung in der Eurotransplant-Region nicht zur Standarddiagnostik zur Beurteilung von Spenderorganen [30]. Proben für eine histologische Untersuchung können entweder im Rahmen einer PLB („percutaneous liver biopsy“) vor oder als offene Keilbiopsie während der Organentnahme gewonnen werden. Für die Leberbiopsie während der Organentnahme mit histologischer Beurteilung werden in der Literatur Raten von 23–28 % angegeben [18, 31]. Für die PLB vor Organentnahme ergab eine kürzlich veröffentlichte Umfrage unter Teilnahme von 58 verschiedenen Organbeschaffungsorganisationen („organ procurement organizations“, OPOs) Raten von nur 5–10 % [32]. Die Autoren kamen zu dem Schluss, dass „zusätzliche Informationen über den Nutzen, die Genauigkeit und die Sicherheit der PLB benötigt werden, um ihren Einsatz zu optimieren“.

Zusammenfassend sind der Stellenwert und die Sicherheit der PLB, ihre Auswirkungen auf die Organallokation, das Transplantationsergebnis und die Kosten bei der Lebertransplantation weiterhin unklar. Ziel unserer Studie war es daher, die Sicherheit der perkutanen PLB zu evaluieren, ihren Einfluss auf die Allokation von Lebertransplantaten hirntoter Spender zu analysieren und mögliche Auswirkungen auf wirtschaftliche und logistische Aspekte bei der Lebertransplantation zu veranschaulichen.

## Material und Methoden

### Studiendesign

Es handelt sich um eine retrospektive Beobachtungsstudie. Bei 36 hirntoten Spendern wurde eine PLB auf der Intensivstation vor geplanter Organentnahme durchgeführt.

### Spenderevaluation und Datenerhebung

Alle ultraschallgesteuerten PLB wurden von einem in der Sonographie erfahrenen (DEGUM-Zertifikat) Transplantationschirurgen mit einer 18-Gauge-Tru-Cut-Nadel durchgeführt, entsprechend unserer zuvor berichteten Technik [33]. Die Thrombozytenzahl war in allen Fällen >50/nl und der Quick-Wert >50 %. Die Gefrierschnitte wurden mit Hämatoxylin- und Eosinfärbung für die histologische Analyse durch einen erfahrenen Pathologen vorbereitet. Die Proben wurden auf Steatose (<30 % = mild, 30–40 % = moderat, >40 % = schwer; makrovesikulär vs. mikrovesikulär), Fibrose, Cholestase, Entzündung, Nekrose und andere Leberpathologien untersucht.

Durch die Deutsche Stiftung Organtransplantation (DSO) erfolgte nach der Feststellung des Hirntodes die Spenderevaluation. Diese bestand aus der Anamnese, den Basisspenderinformationen (Alter, Geschlecht, BMI), Laboruntersuchungen mit Virenscreening und einer Ultraschalluntersuchung der potenziellen Spenderleber. Die Sonographien wurden durch einen erfahrenen Transplantationschirurgen/Intensivmediziner durchgeführt. Ein 5‑MHz-Konvexschallkopf wurde zur Ultraschallbeurteilung der Lebersteatose verwendet („Kidney-Test“), die als „mild“ (erhöhte Echogenität der Leber gegenüber dem Nierenkortex), „moderat“ (Kriterien mild plus Unschärfe der intrahepatischen Gefäße) oder „schwer“ (Kriterien moderat plus abgeschwächte Darstellung der posterioren Lebersegmente und des Zwerchfells, „white liver“) graduiert wurde [26].

Anschließend wurde das Spenderorgan bei der Entnahme durch den Transplantationschirurgen makroskopisch beurteilt. Die Qualität des Spenderorgans wurde als „gut“, „akzeptabel“ oder „schlecht/fett“ eingestuft.

Alle Daten wurden aus den Spenderberichten und medizinischen Aufzeichnungen der 36 hirntoten Spender entnommen.

### Umfragedesign

Es wurde eine Umfrage unter Teilnahme von 11 Transplantationsexperten durchgeführt, um den Bedarf einer PLB und ihre Auswirkungen auf die Allokation zu bewerten. Hieraus ergaben sich 396 virtuelle Fälle (11 Experten × 36 hirntote Spender). Die Umfrage bestand aus drei Stufen. Mit jeder Stufe erhielten die Experten zusätzliche Informationen zum Spender:

- Stufe 1: Anamnese, Basisinformationen zum Spender, Laborergebnisse, Ergebnis der Ultraschalluntersuchung;
- Stufe 2: Ergebnis der makroskopischen Einschätzung des Spenderorgans;
- Stufe 3: histologisches Ergebnis der Leberbiopsie (Tab. 1).

**Table 1:** 

Stufe der Umfrage	Erhältliche Spenderinformationen
Stufe 1	Anamnese
	Basisinformationen (Alter, Geschlecht, BMI)
	Laborbefunde
	Ergebnis Ultraschall
Stufe 2	Makroskopische Bewertung
Stufe 3	Histologisches Ergebnis der Leberbiopsie

*BMI* Body-Mass-Index

**Figure 1:**
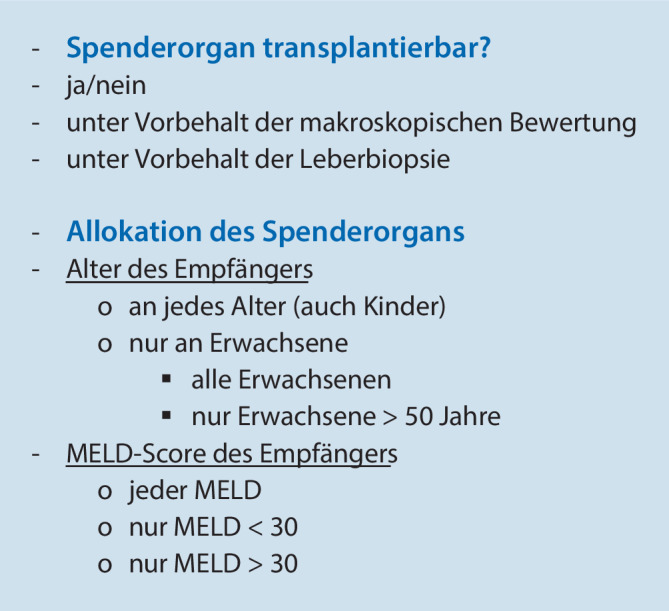

Bei jeder Stufe mussten die Transplantationsexperten bewerten, ob das Spenderorgan transplantierbar ist und für welche Empfänger das Transplantat geeignet wäre (Alter: alle Altersgruppen einschließlich Kinder, nur Erwachsene, alle Erwachsenen, nur Alter >50 Jahre; MELD: alle MELD, nur MELD >30 oder nur MELD <30, Abb. 1) oder ob sie weitere Informationen benötigen würden, um ihre Entscheidung zu treffen.

### Kostenberechnung

Relevante Kostenfaktoren der Lebertransplantation (LT) wurden von der Deutschen Stiftung Organstransplantation (DSO) bezogen und auf der Grundlage der bereitgestellten Daten berechnet.

### Auswertung und statistische Analyse

Die Auswertung der Umfrage bestand aus a) der Häufigkeit der Anforderung weiterer Spenderinformationen; b) dem Einfluss der Spenderinformationen auf die Allokation und auf die Auswahl geeigneter Empfänger; c) der Bewertung der Spendermerkmale, die die Entscheidungsfindung beeinflussen.

Zum Vergleich kategorialer Variablen wurde der exakte Fisher-Test verwendet, zum Vergleich normalverteilter Variablen wurde ein ungepaarter t‑Test mit der Welch-Korrektur verwendet. Die Ergebnisse werden in Mittelwert ± Standardabweichung oder Median und Interquartilsbereich angegeben. *p*-Werte <0,05 wurden als signifikant angesehen. Die Statistiken wurden mit Microsoft Excel 2010 (Microsoft Corporation, Washington) und GraphPad Prism 4 (GraphPad Software, Inc.) berechnet.

## Ergebnisse

### Komplikationen der PLB

Es wurden keine Komplikationen im Zusammenhang mit der PLB beobachtet, d. h. keine intra- oder extrahepatischen Hämatome, keine Galleleckagen oder andere Verletzungen, die die Spenderleber beeinträchtigten. Es kam zu keinem Transplantatverlust durch die PLB.

### Merkmale der Spender, makroskopische Bewertung, Ultraschallergebnisse, Histologie, Korrelation der diagnostischen Tests

#### Merkmale der Spender und Vorliegen erweiterter Spenderkriterien (Abb. 2)

Die Todesursachen der 36 hirntoten Spender waren: subarachnoidale Blutung bei 14 Spendern (39 %), intrakranielle Blutungen bei 8 (22 %), Apoplex bei 7, traumatische Hirnverletzung bei 4 (11 %) und zerebrale Ischämie bei 3 (8 %).

**Figure 2:**
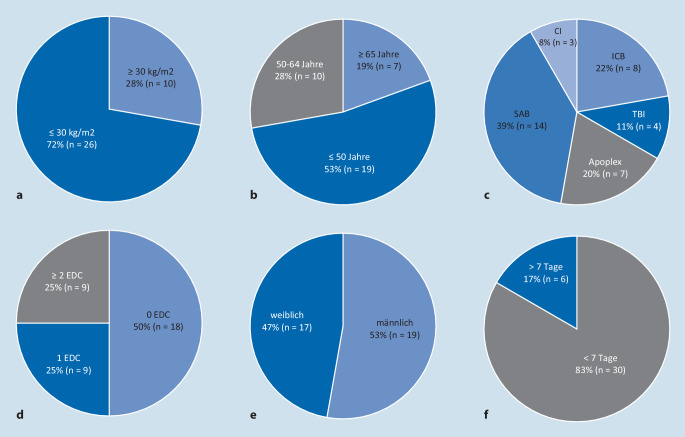

Das mittlere Spenderalter betrug 49,2 ± 17,4 Jahre, wobei 19 Spender <50 Jahre (52 %), 10 Spender 50 bis 64 Jahre (28 %) und 7 >65 Jahre (19 %) alt waren. 19 waren männlich (53 %) und 17 weiblich (47 %). Der durchschnittliche BMI betrug 27,2 ± 4,7 kg/m^2^, mit 26 Spendern <30 kg/m^2^ (72 %) und 10 Spendern >30 kg/m^2^ (28 %). Die durchschnittliche Aufenthaltsdauer auf der Intensivstation betrug 5,3 ± 4,8 Tage. 30 Spender hatten einen Intensivaufenthalt von <7 Tagen (83 %) und 6 Spender >7 Tage (17 %). Die Laborergebnisse der Spender zeigten eine mittlere Serum-Aspartat-Aminotransferase (AST) von 480 nmol/(s l) ± 326 nmol/(s l) ohne eine Erhöhung über 1500 nmol/(s l). Die mittlere Serum-Alanin-Aminotransferase (ALT) betrug 683 nmol/(s l) ± 400 nmol/(s l), bei 2 Spendern lagen die Werte ≥ bei 1500 nmol/(s l) (6 %). Der mittlere Serumbilirubinwert betrug 25,6 µmol/l ± 34 µmol/l, bei 4 Spendern waren Werte >3 mg/dl (11 %) zu verzeichnen. Insgesamt 18 Spender hatten keine EDC (50 %), 9 hatten 1 EDC (25 %) und 9 hatten ≥2 EDC (25 %).

#### Ultraschallergebnisse

**Figure 3:**
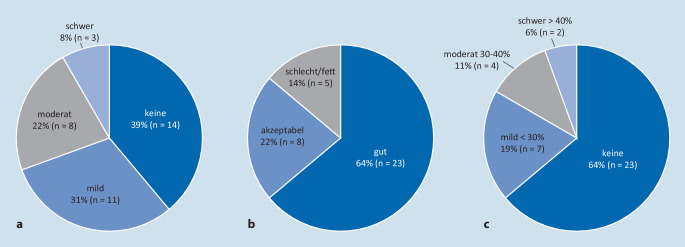

Die Sonographie bewertete 14 Spenderorgane (39 %) als „keine Steatose“, 11 (31 %) als „milde Steatose“, 8 (22 %) als „moderate Steatose“ und 3 (8 %) als „schwere Steatose“ (Abb. 3; Tab. 2).

**Table 2:** 

	Gut (keine Steatose oder <30 %: mild)	Akzeptabel (Steatose 30–40 %: moderat)	Schlecht (Steatose >40 %: schwer)
*Ultraschall*	25 (69 %)	8 (22 %)	3 (8 %)
*Makroskopische Bewertung*	23 (64 %)	8 (22 %)	5 (14 %)
*Leberbiopsie*	30 (83 %)	4 (11 %)	2 (6 %)

#### Makroskopische Bewertung während der Organentnahme

Bei der makroskopischen Beurteilung der Spenderlebern durch den Transplantationschirurgen während der Organentnahme wurden 23 (64 %) als „gut“, 8 (22 %) als „akzeptabel“ und 5 (14 %) als „schlecht/fett“ bewertet (Abb. 3; Tab. 2).

#### Histologische Ergebnisse der PLB

Die histologische Untersuchung der Leberbiopsien ergab bei 21 der 36 Spender (58 %) den Befund einer Steatose, davon bei 13 (36 %) eine Makrosteatose (Tab. 3). Die Makrosteatose wurde bei 2 Spendern (6 %) als „schwer“ (Steatose >40 %), bei 4 (11 %) als „moderat“ (Steatose 30–40 %) und bei 7 (19 %) als „mild“ (Steatose <30 %) eingestuft (Abb. 3; Tab. 2). Eine Makrosteatose >30 % war bei Spendern mit BMI ≥30 kg/m^2^ häufiger als bei Spendern mit BMI <30 kg/m^2^, aber zwischen den BMI-Gruppen statistisch nicht signifikant (*p* = 0,7).

**Table 3:** 

Pathologie	Alle Spender (*n* = 36)	Spender BMI <30 kg/m^2^ (*n* = 26)	Spender BMI ≥30 kg/m^2^ (*n* = 10)
*Steatotisch; n (%)*			
Makrosteatose 40 %	2 (5,5)	1 (3,8)	1 (10,0)
Makrosteatose 30 %	4 (11,1)	3 (11,5)	1 (10,0)
Makrosteatose <20 %	7 (11,1)	4 (15,4)	3 (30,0)
Mikrosteatose	13 (30,5)	8 (30,8)	5 (50,0)
Gemischte Steatose	9 (22,2)	4 (15,4)	5 (50,0)
*Nichtsteatotisch; n (%)*			
Vakuolardegeneration	1 (2,8)	1 (3,8)	–
Unspezifischer Hepatozytenschaden	1 (2,8)	1 (3,8)	–
Leichtgradige Fibrose	2 (2,8)	1 (3,8)	1 (10,0)
Zellschwellung	1 (2,8)	1 (3,8)	–
Cholestase	6 (16,6)	2 (7,6)	4 (40,0)
Periportale Entzündung	1 (2,8)	–	1 (10,0)

*BMI* Body-Mass-Index

Andere, nichtsteatotische Pathologien (leichtgradige Fibrose, Cholestase, unspezifischer Hepatozytenschaden, Vakuolardegeneration, periportale Entzündung, Zellschwellung) wurden bei 10 Spendern (28 %) diagnostiziert (Tab. 3).

#### Korrelation von Ultraschall und Histologie

Sonographisch wurden 3 Spenderorgane als „schwere“ Lebersteatose eingestuft. Histologisch zeigten davon 2 Leberbiopsien eine „schwere“ (40 % Makrosteatose) und 1 eine „moderate“ Steatose (30 % Makrosteatose). Von den 8 sonographisch als „moderat“ steatotisch eingestuften Lebern wiesen 4 histologisch keine Steatose auf.

Insgesamt wurden sonographisch 25 Spenderorgane als „keine Steatose“ oder „mild“ steatotisch klassifiziert. Unter diesen lag histologisch nur in einem Fall eine „moderate“ Makrosteatose vor.

Hieraus resultierten eine Sensitivität und Spezifität des Ultraschalls zur Erkennung einer „moderaten“ und „schweren“ Makrosteatose ≥30 % von 83 bzw. 80 % (Tab. 4).

**Table 4:** 

	Leberbiopsie: „moderat“ oder „schwer“	Leberbiopsie: „keine“ oder „mild“	Total
**Ultraschall: „moderat“ oder „schwer“**	5 *(richtig-positiv)*	6 *(falsch-positiv)*	11
**Ultraschall: „keine“ oder „mild“**	1 *(falsch-negativ)*	24 *(richtig-negativ)*	25
**Total**	6	30	36

#### Korrelation von makroskopischer Bewertung und Histologie

Insgesamt 13 Spenderlebern wurden makroskopisch als „akzeptabel“ oder „schlecht/fett“ eingestuft. Unter diesen lag histologisch in 6 Fällen eine „moderate“ oder „schwere“ Makrosteatose ≥30 % vor, 7 wiesen „keine“ oder eine „milde“ Makrosteatose auf.

Von den 23 makroskopisch als „gut“ bewerteten Spenderlebern zeigte histologisch keine eine „moderate“ oder „schwere“ Makrosteatose ≥30 %.

Sensitivität und Spezifität der makroskopischen Bewertung zur Detektion einer „moderaten“ oder „schweren“ Makrosteatose ≥30 % lag damit bei 100 bzw. 77 % (Tab. 5).

**Table 5:** 

	Leberbiopsie: „moderat“ oder „schwer“	Leberbiopsie: „keine“ oder „mild“	Total
**Makroskopische Bewertung: „moderat“ oder „schwer“**	6 *(richtig-positiv)*	7 *(falsch-positiv)*	13
**Makroskopische Bewertung: „keine“ oder „mild“**	0 *(falsch-negativ)*	23 *(richtig-negativ)*	23
**Total**	6	30	36

### Umfrage

#### Bedarf einer makroskopischen Bewertung

**Figure 4:**
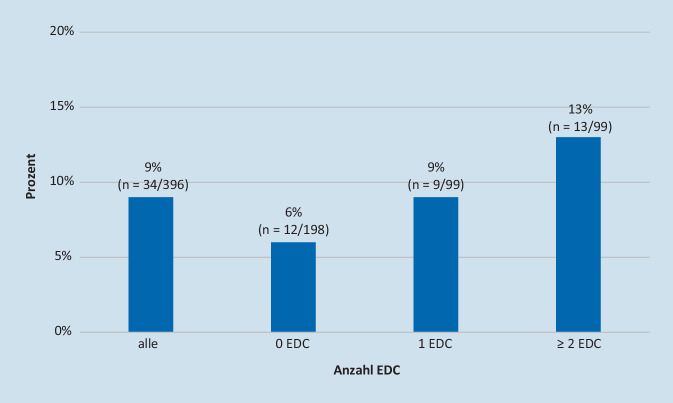

Eine makroskopische Bewertung wurde in 9 % der Fälle gefordert. Die Nachfrage einer makroskopischen Bewertung stieg parallel zur Anzahl der vorliegenden EDC: 6 % ohne EDC, 9 % mit 1 EDC und 13 % mit ≥ 2EDC (Abb. 4).

#### Änderungen der Allokation durch die makroskopische Bewertung

**Figure 5:**
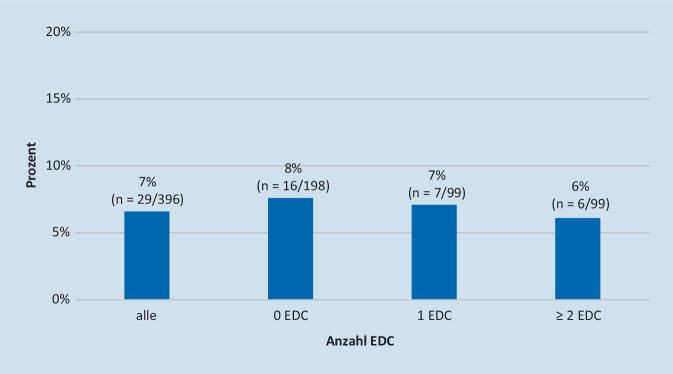

Die makroskopische Bewertung führte in 7 % aller Fälle zu einer Änderung der Allokation. Es gab keine signifikanten Unterschiede zwischen Spendern mit und ohne EDC (Abb. 5). In 69 % der Fälle, die zu einer Änderung der Allokation führten, verlangten die Experten zunächst keine makroskopische Bewertung.

#### Bedarf einer Leberbiopsie mit Histologie

**Figure 6:**
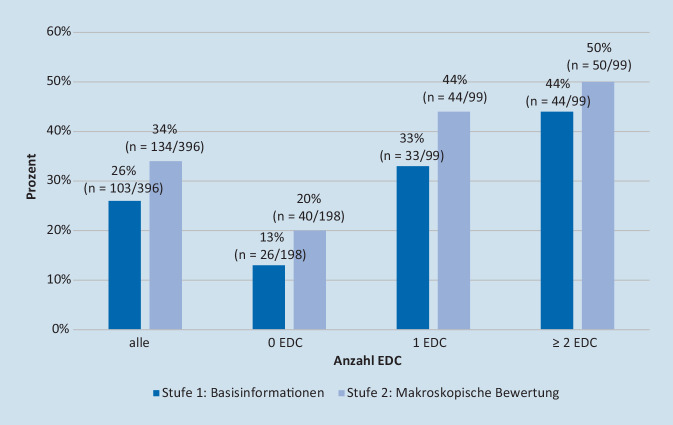

Eine Leberbiopsie wurde in 26 % der Fälle nach Stufe 1 (Basisinformationen zum Spender) verlangt. Die Nachfrage stieg nach Schritt 2 (makroskopische Bewertung) auf 34 %. Darüber hinaus stieg die Nachfrage nach einer Leberbiopsie signifikant mit der Anzahl der EDC an, mit einem Maximum von 50 % bei Spendern mit ≥2 EDC (Abb. 6).

**Figure 7:**
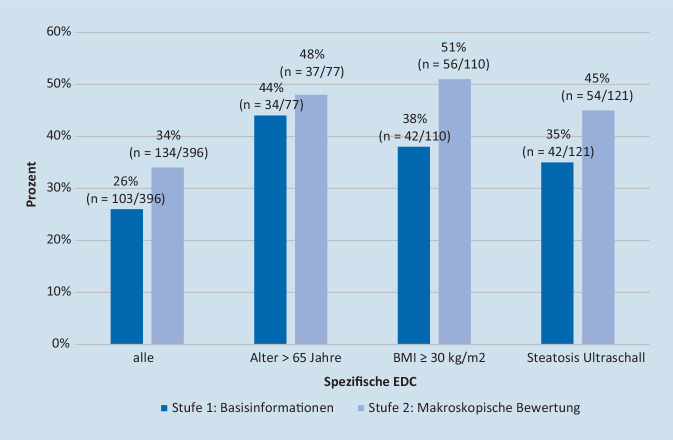

Eine Subgruppenanalyse 3 verschiedener EDC (Alter >65 Jahre; BMI ≥30 kg/m^2^; Steatose bei Ultraschall) und die damit einhergehende spezifische Häufigkeit der Anforderung einer Leberbiopsie ist in Abb. 7 dargestellt. Ein Alter >65 Jahre wurde als das EDC mit der höchsten Nachfrage nach einer Leberbiopsie nach Stufe 1 (44 %) identifiziert, ein BMI ≥30 kg/m^2^ ergab die höchste Nachfrage nach Leberbiopsie nach Schritt 2 (51 %).

#### Änderung der Allokation durch die Leberbiopsie

**Figure 8:**
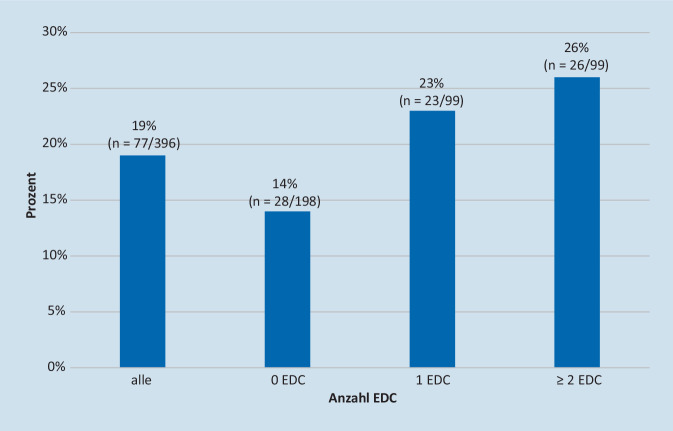

Eine Leberbiopsie führte in 19 % aller Fälle zu einer Änderung der Allokation. Diese nahm mit der Anzahl der EDC zu: 14 % Änderungen der Allokation bei Spendern ohne EDC, 23 % mit 1 EDC und 26 % mit ≥2 EDC (Abb. 8). In 50 % der Fälle mit Änderung der Allokation nach Leberbiopsie gaben die Experten initial an, diese nicht zu benötigen.

### Kostenberechnung

Daten zur Kostenberechnung im Zusammenhang mit der Organentnahme und der Allokation wurden von der Deutschen Stiftung Organtransplantation (DSO) zur Verfügung gestellt. Im Fall einer unnötigen Organentnahme mit anschließendem Transport, bei dem die Leber aufgrund des histologischen Ergebnisses letztendlich verworfen werden muss, ergeben sich Kosten (Personal, Logistik und Material) zwischen 4685 € (Bodentransport) und 10.895 € (Lufttransport). Im Falle einer bereits veranlassten stationären Aufnahme eines designierten Transplantatempfängers mit präoperativer Vorbereitung (Labordiagnostik, Röntgen, Vorbereitung der Operation und Transplantationsteam) fallen zusätzliche Kosten von mindestens 2500 € an.

Insgesamt 491 Lebertransplantate, die in Deutschland von 2010 bis 2016 alloziert wurden, wurden schließlich nicht transplantiert [34]. Dies ergibt durchschnittlich 70 unnötige Organentnahmen und Allokationen jährlich. Diese Zahl muss mit den oben genannten Kosten multipliziert werden, um das Einsparungspotenzial in Deutschland pro Jahr durch eine PLB vor Organentnahme zu berechnen.

## Diskussion

In Deutschland bestehen bei der Beurteilung der Organqualität von Spenderlebern weiterhin regelmäßig Diskrepanzen zwischen sonographischer und makroskopischer Einschätzung einerseits und dem histologischen Befund, welcher in der Regel erst vorliegt, nachdem die Allokation bereits gestartet wurde. Mögliche Folgen sind ein „mismatch“ zwischen Lebertransplantat und Empfänger sowie eine nachträgliche Änderung der Allokation mit Verlängerung der kalten Ischämiezeit.

Ziel unserer Studie war es, die Sicherheit der PLB vor Organentnahme auf Intensivstation und ihre Auswirkungen auf Allokation und Kosten bei der LT zu bewerten.

Es traten keine Komplikationen durch die ultraschallgesteuerte PLB auf, womit diese sich als sicheres invasives diagnostisches Verfahren erwies, was durch die Berichte von Savas et al. [9], Nadalin et al. [33] sowie Ryan et al. [35] untermauert wird. In den zitierten Studien traten bei potenziellen Leberlebendspendern in 201, 144 und 100 durchgeführten PLB keine Komplikationen auf. Aktuelle Daten berichten über Komplikationsraten nach PLB um 0,57 %, und Komplikationen steigen mit der Anzahl der Biopsieversuche [36]. Bezüglich der Mindestqualifikation des Biopsierenden sahen Westheim et al. eine erhöhtes postinterventionelles Blutungsrisiko bei vorliegender Erfahrung von <20 Biopsien (3,2 %) gegenüber >100 Biopsien (1,1 %; [37]). Bei einem Durchmesser der Biopsienadel >1,6 mm kann es ebenfalls zu vermehrten Blutungskomplikationen kommen [38]. Aus diesem Grund empfehlen wir folgende Standards für die PLB: ultraschallgesteuerte Biopsie, vorhandene Erfahrung des Durchführenden in Form von mindestens >20 Biopsien, nicht mehr als ein Biopsieversuch und Verwendung einer 18-Gauge-Tru-Cut-Nadel [39].

Unsere Ergebnisse zeigten eine nicht ausreichend zuverlässige Korrelation zwischen Ultraschallbefunden und makroskopischer Bewertung mit den Ergebnissen der Leberbiopsie in Bezug auf das Vorliegen sowie die Gradierung einer Steatose. Für die Leberbiopsie konnten wesentliche Vorteile zur Beurteilung von Spenderlebern demonstriert werden. Sowohl die Ultraschalluntersuchung als auch die makroskopische Bewertung stuften die Spenderlebern in 20 und 23 % der Fälle als „moderate“ oder „schwere“ Makrosteatose ein, während histologisch nur eine „geringe“ oder gar „fehlende“ Makrosteatose vorlag. Andere Studien zeigten eine noch geringere Korrelation zwischen Ultraschall [9], makroskopischer Bewertung [40] und histologischen Ergebnissen. Dies kann dazu führen, dass Spenderorgane aufgrund der sonographischen oder makroskopischen Einschätzung fälschlicherweise verworfen werden, insbesondere da eine sonographisch diagnostizierte Lebersteatose als ein Hauptgrund für die Verwerfung von Spenderorganen gilt [41]. Czerwinski et al. zeigten, dass 35 % der verworfenen Lebertransplantate bei der abschließenden histologischen Untersuchung nur minimale Veränderungen aufwiesen und somit zur Transplantation hätten verwendet werden können [42]. Die histologische Untersuchung ist nach wie vor der Goldstandard für den Nachweis einer Steatose und anderen Leberpathologien wie NASH, Fibrose, Entzündung, Cholestase oder unspezifischer Hepatitis [24], welche allesamt ebenfalls eine Kontraindikation für eine Transplantation darstellen können.

Unsere Umfrage zeigte, dass die PLB eine entscheidende Rolle in der Allokation spielt, bei der sie in 19 % der Fälle zu einer Änderung führte. Bei Vorhandensein von EDC stieg der Einfluss der PLB auf die Allokation weiter an (23 % bei Spendern mit 1 EDC, 26 % in Fällen mit ≥2 EDC), was ihren Stellenwert insbesondere bei ECD-Spendern unterstreicht. Interessanterweise verlangten die Experten in fast 50 % der Fälle mit Änderung der Allokation nach Erhalt des histologischen Befundes zunächst keine Leberbiopsie.

Im Gegensatz dazu führte die makroskopische Bewertung nur in 7 % aller Fälle zu einer Änderung der Allokation, ohne dass es einen signifikanten Unterschied zwischen der Anzahl an vorliegenden EDC gab.

Yersiz et al. [43] zeigten, dass eine makrovesikuläre Steatose vom Transplantationschirurgen anhand der Lebermorphologie (Gelbfärbung, abgerundete Kanten) mit einer Genauigkeit von bis zu 86 % vorhergesagt werden kann. Für eine makroskopische Graduierung der Steatose sank die Genauigkeit jedoch auf nur 75 %. Unter anderem auf Grundlage dieser Ergebnisse haben die Deutsche Transplantationsgesellschaft, die Deutsche Stiftung Organtransplantation und die Deutsche Gesellschaft für Pathologie kürzlich Indikationen für die Gewinnung einer Leberbiopsie während der Organentnahme definiert (Schätzung der Lebersteatose durch den Transplantationschirurgen ≥30 %; [30]).

Eine PLB bietet im Vergleich zur offenen Biopsie während der Organentnahme mehrere Vorteile, mit entsprechend hohem Potenzial zur Verbesserung des Outcomes bei der Lebertransplantation. Das histologische Ergebnis der PLB liegt bereits vor geplanter Organentnahme vor. Hierdurch kann die Auswahl eines idealen Transplantatempfängers in einem frühen Stadium des Allokationsverfahrens erfolgen und eine Änderung der Allokation mit damit einhergehender Verlängerung der kalten Ischämiezeit vermieden werden. Eine lange kalte Ischämiezeit ist mit schlechteren Ergebnissen bei Lebertransplantationen assoziiert, insbesondere bei Spenderorganen mit einer Makrosteatose >15 % [44, 45] sowie bei Transplantaten älterer Spender [4]. Oliver et al. zeigten kürzlich eine signifikant niedrigere Komplikationsrate in einer ECD-Kohorte mit PLB im Vergleich zu einer Kohorte ohne PLB (31,9 vs. 42,3 %; *p* < 0,001; [46]). Der positive Einfluss einer standardmäßigen Leberbiopsie auf das Transplantationsergebnis wurde auch schon früher von Markin und Kollegen demonstriert [47]. In dieser Studie wurde die primäre Nichtfunktionsrate von Spenderlebern nach Einführung der PLB von 8,5 auf 1,4 % reduziert.

Darüber hinaus können durch eine PLB unnötige Verwerfungen von Lebertransplantaten vermieden werden, mit damit einhergehender Erweiterung des Pools dringend benötigter Spenderorgane. Bei histologisch diagnostizierter Kontraindikation für eine Transplantation können wiederum unnötige Organentnahmen vermieden werden [46]. Wird eine potenzielle Spenderleber mittels PLB als „marginal“ identifiziert, könnte in ausgewählten Fällen eine Vorkonditionierung (durch maschinelle Perfusion) zur Verbesserung der Transplantatqualität durchgeführt werden [48]. Eine PLB wird in der Regel tagsüber während der regulären Arbeitszeiten durchgeführt, was eine bessere Kommunikation zwischen allen Beteiligten (Pathologe, Transplantationschirurg, DSO) ermöglicht.

**Figure 9:**
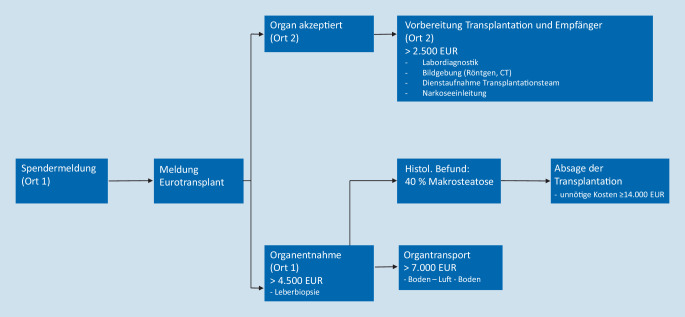

Auf der Grundlage der von der Deutschen Stiftung Organtransplantation bereitgestellten Daten konnten wir potenzielle Kosteneinsparungen durch eine PLB für verschiedene Szenarien abschätzen. Eine genaue Berechnung des Einsparungspotenzials erscheint jedoch schwierig, da dieses von einer Vielzahl verschiedenster Faktoren abhängt, welche sich auf die tatsächlichen Kosten auswirken. Nach unserer Kenntnis hat sich bislang keine Studie mit den möglichen wirtschaftlichen Aspekten der PLB befasst. Hierzu zeigen die Abb. 9 und 10 verschiedene Szenarien mit und ohne PLB, jeweils mit möglichen Auswirkungen auf die Kosten während der Allokation. Zusammenfassend belaufen sich die potenziellen Kosteneinsparungen für eine vermeidbare Organentnahme mit anschließendem Organtransport auf bis zu 14.000 €.

**Figure 10:**
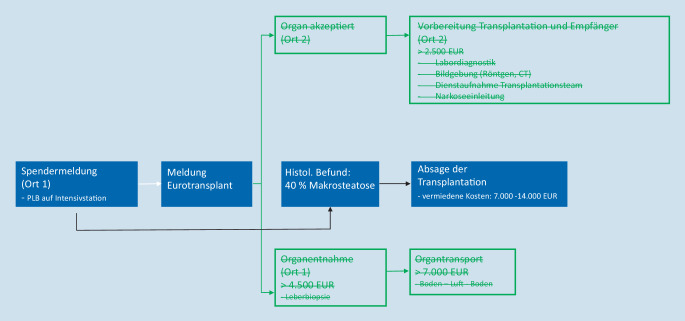

## Schlussfolgerung

Anhand der Ergebnisse unserer Studie konnten wir zeigen, dass eine PLB 1) sicher ist, 2) einen hohen Einfluss auf die Allokation bereits vor der Organentnahme hat (insbesondere bei Spendern mit EDC) und damit in vielen Fällen die kalte Ischämiezeit reduzieren kann, 3) das „matching“ zwischen Spender und Empfänger optimiert und 4) ein hohes Kosteneinsparungspotenzial besitzt. Unsere Daten unterstreichen damit die hohe klinische und ökonomische Relevanz der PLB im Rahmen der Lebertransplantation. Unserer Meinung nach sollte eine PLB bei allen potenziellen Spendern, vor allem aber bei solchen mit EDC durchgeführt werden.

## Abkürzungen

ALT: Alanin-Aminotransferase
AST: Aspartat-Aminotransferase
BAR: Balance of risk (score)
BDD: Brain-dead donors, hirntote Spender
BMI: Body-Mass-Index
CI: Zerebrale Ischämie
CIT: Cold ischemia time, kalte Ischämiezeit
CT: Computertomographie
DEGUM: Deutsche Gesellschaft für Ultraschall in der Medizin
DRI: Donor risk index
DSO: Deutsche Stiftung Organtransplantation
ECD: Extended criteria donors, Spender mit erweiterten Spenderkriterien
EDC: Extended donor criteria, erweiterte Spenderkriterien
ET: Eurotransplant
FLD: Fatty liver disease, Fettlebererkrankung
GGT: Gamma-Glutamyltransferase
ICB: Intrakranielle Blutung
ICU: Intensive care unit, Intensivstation
LT: Lebertransplantation
MELD: Model of end stage liver disease
NASH: Non-alcoholic steatohepatitis, nichtalkoholische Hepatitis
OLT: Orthotopic liver transplantation, orthotope Lebertransplantation
OPO: Organ procurement organization
PLB: Percutaneous liver biopsy
PNF: Primary non function
SAB: Subarachnoidalblutung
TBI: Traumatic brain injury, traumatischer Hirnschaden
USD: US-Dollar
